# Wellbeing and national identity in three generations of Czech and Slovak Holocaust survivors

**DOI:** 10.3389/fnbeh.2022.919217

**Published:** 2022-09-05

**Authors:** Marek Preiss, Monika Fňašková, Markéta Nečasová, Radek Heissler, Petr Bob, Alice Prokopová, Dita Šamánková, Edel Sanders, Ivan Rektor

**Affiliations:** ^1^National Institute of Mental Health (Czechia), Prague, Czechia; ^2^Central European Institute of Technology (CEITEC), Brno, Czechia; ^3^Department of Psychology, University of New York in Prague, Prague, Czechia

**Keywords:** trauma, PTSD, transgenerational, Czech, Holocaust

## Abstract

Subjective wellbeing (SWB) is an important factor of global adjustment. Intergenerational satisfaction in seriously traumatized people has not been studied so far in homogenous populations of Central and Eastern Europe. This study focuses on the SWB in three generations of survivors living in the Czech Republic and Slovakia after World War II (WWII). The focal groups were Holocaust survivors (ages 71–95, *n* = 47), Holocaust survivors’ children (ages 30–73, *n* = 86), and their grandchildren (ages 15–48, *n* = 88), and they were compared to aged-matched groups without Holocaust history. The first and second generation of Holocaust survivors scored significantly lower than the comparison groups in wellbeing, as measured using the Schwartz Outcome Scale-10 (SOS-10). There was no significant difference in life satisfaction in any of the three generations. Within the focal group, identification as Jewish or as also Jewish was comparable in all three generations of Holocaust survivors (74% in the first, 79% in the second, and 66% in the third generation). Holocaust survivors declaring Jewish identity reported lower SWB compared to survivors declaring other than Jewish identity. The focal group generated more national identities than comparisons. The outcomes are discussed in the context of the history of Central and Eastern Europe.

## Introduction

Subjective wellbeing (SWB) is a multifaceted phenomenon with both affective and cognitive aspects (Morrison et al., 2011) that refers to positive feelings about one’s life and one’s self (Watson et al., 2010). The construct whose elements transcend solely economic prosperity includes people’s emotional responses, domain satisfactions (work, family, leisure, health, finances, self, and one’s group), and global judgments of life satisfaction (Diener et al., 1999). SWB operates in both private (experiential SWB) and public areas (declarative SWB) at synchronic (differential SWB) and diachronic (narrative SWB) trajectories of interaction (Shmotkin et al., 2006).

Subjective wellbeing has been investigated within psychology and other social and natural sciences (Kebza, 2005) mainly to evaluate the process of personal development (e.g., Bornstein et al., 2003) and treatment outcomes (e.g., Pettersson and Bergbom, 2019). Adjustment and wellbeing can be seriously affected by traumatic events, such as natural and man-made disasters or historical traumas. From the perspective of the World War II (WWII) aftermath, it is important to study not only survivors’ adjustment and wellbeing but also their transmission of trauma onto the generations of their offspring (Shrira et al., 2011; Danieli et al., 2016; Greenblatt-Kimron et al., 2021; Greenfeld et al., 2022).

Adverse or traumatic events to which parents have been exposed can have a profound psychological effect on their descendants in different populations (e.g., Fridman et al., 2011), and the Holocaust is one of the most researched examples of transgenerational transmission of trauma. Trauma transmission is typically examined from different perspectives: psychodynamic, family systems, epidemiological, sociological, and biological. The impact of trauma like the Holocaust is transmitted through the ruptures in the community and changes in identity (Lehrner and Yehuda, 2018). Traumatic symptoms of parents can result in similar symptoms among second-generation individuals who were not exposed to the Holocaust trauma or did not hear about it (Cohn and Morrison, 2017). Wellbeing can be an example of a psychological variable that, in addition to current and dispositional variables, is also a reflection of phenomena transmitted through generations, including trauma (Weinberg and Cummins, 2013).

Barel et al. (2010) measured Holocaust survivors’ wellbeing defined as a general social adaptation or personality development by the Tennessee Self-Concept Scale (Fitts, 1971) and other instruments in a set of 26 non-select samples (adequately designed studies, *N* = 7,367). This meta-analysis showed a significant difference in psychological wellbeing between Holocaust survivors and comparisons, with a combined effect size of *d* = 0.14, *p* < 0.050). Similarly, survivors found less satisfaction with their lives (Fridman et al., 2011).

As for the first, second, and third generations, two recent meta-analyses (Sagi-Schwartz et al., 2003; van IJzendoorn et al., 2003) indicated that the Holocaust survivors’ offspring did not differ significantly from comparisons on various measures of psychological wellbeing. Sagi-Schwartz et al. (2003) did not find a significant difference in psychological wellbeing between the third generation of Holocaust survivors and their comparisons in a sample of more than 1,000 respondents from 13 subgroups. van IJzendoorn et al. (2003) did find a significant difference in psychological wellbeing, defined as a combination of general mental health and adaptation measured with the Brief Mental Health Index (Gunderson and Arthur, 1969) and Tennessee Self-Concept Scale (Fitts, 1965), between second-generation Holocaust survivors and their comparisons in a sample of more than 4,400 subjects of 32 subgroups. The difference, however, was significant only in select, randomized samples.

Barel et al. (2010) suggested that some factors, such as the country of residence, could modify the impact of the secondary trauma among the Holocaust survivors’ descendants. In their international study, poorer wellbeing was reported by the Holocaust survivors living in countries other than Israel based on the hypothesis that one’s country of residence can serve as a moderator for psychological wellbeing. van IJzendoorn et al. (2003, p. 467), on the other hand, suspect that “Israel as a symbol of survival may not sufficiently have materialized its potentially protective role.”

A nation or nationality is traditionally outlined by a common language, tradition, and culture that is more or less fluid and rather socially and culturally (as opposed to biologically) conditioned (Jenkins, 2008), while ethnicity is usually considered to be a primordial set of inheritable features (King, 2001). These two concepts historically have been often comprehended as identical regarding ethnicity rather than as a genetic base for the development of cultural characteristics. Some authors (e.g., Anderson, 1991) understand nationality as a mere political construct.

Jewish identity or Jewishness defies proper definition in any of the existing frames of nationality and/or ethnicity since it comprises many elements of tradition, language, religion, shared history or “destiny,” “exclusivity,” and “special character features,” some of which might be completely missing in an individual (e.g., language, religion) without diminishing their sense of Jewishness (Čapková, 2014). Biale (2002) offers a view on Jewish identity, conceiving different Jewish cultures (in plural) in different times and geopolitical conditions *via* the contextual lens of the continuity of written tradition (the Bible and Talmud) and customs shared by different Jewish cultures throughout the entirety of Jewish history. The ambiguity of Jewishness is, in any case, troublesome in any research where Jewish identity is an important variable.

In the Czech Republic, the reasons for denial of one’s own Jewishness waned after the anti-communist Velvet revolution (a non-violent transition of power in former Czechoslovakia) of 1989. Some of the survivors’ millennial grandchildren, who can study and travel again without restriction, do indeed show a tendency to “overcompensate” the identity loss and/or confusion of their families, keenly seeking and promoting “all things Jewish.” In neither generation, however, the feelings of Jewishness are grounded in Judaism or language (Hebrew or Yiddish), contrary to, for instance, the now large Vietnamese population in the Czech Republic, who keep their national identity mainly through language (Hubertová, 2016). Rather, the Jews express their Jewishness as a “sense of belonging to the past and future generations of Jews” (Heitlinger, 2006, p. 199).

As one analysis of the questionnaire data determined, Jewish identities “can be thin or thick, weak or strong, virtual, symbolic, based on fate, self-defined, externally imposed, secular, religious, hybrid, in transition, linked to several nations, or labeled se émigré, Czechoslovak, Czech, or Slovak” (Heitlinger, 2006, p. 196).

### Current research

The study is a part of a multidimensional project titled “Health and wellbeing of Czech Holocaust survivors and their offspring—the mechanism of transgenerational transfer of trauma.” The Grant by (Czech Health Research Council) was conducted in (Ministry of Education, Youth and Sports of the Czech Republic) during 2016–2019 with the aim to make a three-generational assessment of the Holocaust-related trauma in the Czech environment.

The presented part of the study focuses on three main goals: (1) comparison of the level of wellbeing of the three generations of Holocaust survivors with the general population respondents matched by age; (2) exploration of the national/ethnic identity of the three generations of Holocaust survivors; and (3) comparison of the level of wellbeing of respondents with Jewish vs. non-Jewish national/ethnic identity.

So far, research focused on the wellbeing of Holocaust survivors in relation to their Jewish or other ethnic/national identity has been mostly assessed in samples of war emigrants from Europe to Israel and western countries (e.g., Jaspal and Yampolsky, 2011; Schwartzman, 2015). To the best of our knowledge, very few similar studies have been conducted in the Central/Eastern European territory amongst subjects who stayed in a country that had experienced Nazi persecution during WWII.

## Materials and methods

### Participants and procedure

The total sample consisted of 378 participants (focal group *n* = 221; comparison group *n* = 157). The focal group consisted of three subgroups: Holocaust survivors (n*FG1* = 47), Holocaust survivors’ children (*nFG2* = 86), and grandchildren (*nFG3* = 88) (see Table 1 for details). The subgroups were (with few exceptions) genetically unrelated. The age-matched comparison groups comprised *nCG1* = 31, *nCG2* = 62, and *nCG3* = 64. Groups were not different according to the χ^2^-test of symmetry in gender in neither the first [χ^2^(1, *N* = 78) = 0.56, *p* = 0.450], second [χ^2^(1, *N* = 148) = 0.54, *p* = 0.460], nor the third [χ^2^(1, *N* = 152) = 0.8, *p* = 0.370] generation. The age ranges for the focal groups were 71–95 (G1), 30–73 (G2), and 15–48 (G3); for comparisons 73–90 (G1), 36–73 (G2), and 16–47 (G3). According to the Mann–Whitney *U*-test, there were no differences in age between groups in all generations (all *p* > 0.050) (see Table 1 for details).

**TABLE 1:** Descriptive statistics of the focal and comparison groups.

Focal				Comparison			
Generation	Men (*n*)	Age (*M* ± *SD*)	SOS-10 (*M* ± *SD*)	Generation	Men (*n*)	Age (*M* ± *SD*)	SOS-10 (*M* ± *SD*)
1 (*n* = 47)	16	82.07 ± 6.26	46.74 ± 8.97	1 (*n* = 31)	14	79.84 ± 4.20	52.39 ± 7.42
2 (*n* = 86)	34	60.84 ± 8.78	46.71 ± 9.35	2 (*n* = 62)	20	59.84 ± 9.27	50.08 ± 8.41
3 (*n* = 88)	31	36.66 ± 8.77	43.85 ± 9.53	3 (*n* = 64)	28	33.19 ± 8.34	46.72 ± 8.39

SOS-10, Schwartz Outcome Scale.

All participants were born in Czechoslovakia or its successor states (the Czech Republic and Slovakia) established after its split in 1993 and lived there all their lives, i.e., they shared a similar geopolitical background. Since most of the Czech Holocaust survivors are not registered in any organization (such as Jewish Communities) and it is difficult to get their contact details, volunteers were recruited using a snowball method (by public appeal presented by the University in Brno, national and local Czech media, and by direct addressing through local Jewish Communities). The focal subgroup G1 (Holocaust survivors) were included in the study based on their direct experience with Nazi persecution during WWII (surviving concentration camps or as hidden people) because of being regarded as Jewish according to the Nuremberg Laws; G2 consisted of people who proclaimed that at least one parent was a Holocaust survivor (as defined in G1); and G3 subjects proclaimed that at least one grandparent was a Holocaust survivor (as defined in G1). Most of the participants in subgroups G1, G2, and G3 were not genetically related members of one family.

The comparison group respondents, matched by age, did not have a Holocaust family history, were not exposed to any extreme stress before the investigation, and did not proclaim Jewish ethnicity and/or religion.

Exclusion criteria for all focal and comparison groups included a history of treatment for severe psychiatric disorders (such as psychosis), any kind of severe brain impairment (brain injury, tumors, and neurodegenerative diseases), and significant cognitive decline (all participants scored over 26 points in the Mini-Mental State Examination).

### Ethics

All participants provided their written informed consent before enrolling in the study. The study was conducted in accordance with the Declaration of Helsinki, and the protocol was approved by the University of (removed for blind review). All the data were collected between 2016 and 2019. The still unprocessed biological data not related to the psychological parts of the study, such as biomarkers of chronic stress, genetic impact of extreme stress, and brain mapping, are planned for publication in other types of journals.

### Measures

#### Schwartz Outcome Scale-10

The scale represents a broad construct related to multiple aspects of psychological functioning and psychological wellbeing. An example of an item is “Given my current physical condition, I am satisfied with what I can do.” The 10-item measure was rated by respondents on a 7-point scale ranging from 0 (never) to 6 (all the time or nearly all the time). The questions are focused on physical, psychological, and social functioning regarded as core components of wellbeing. Strong positive correlations were associated with a desire to live, positive self-esteem, positive affect, a sense of coherence, and physical health; strong negative correlations were associated with psychopathology, hopelessness, fatigue, and negative affect (Blais et al., 1999). The total Schwartz Outcome Scale-10 (SOS-10) scores range from 0 to 60. A higher score is associated with greater wellbeing. In the original study (Blais et al., 1999), Cronbach’s alpha was above.90 in three samples. The scale shows no ceiling effect. In a Czech sample of the SOS-10 (Dragomirecka et al., 2006), Cronbach’s alpha was.9; in our sample, McDonald’s ω was.84. The factor analysis replicated the structure of the English version of the tool with one factor, which accounted for 57% of the variance (76% of the variance in the original sample). The scale discriminated well between patients and controls, with patients scoring significantly lower (Dragomirecka et al., 2006).

#### Life satisfaction

Participants were asked two questions: “Are you satisfied with your personal life in a lifelong view?” and “Are you satisfied with your career in a lifelong view?”. Possible responses were: yes—rather yes—something in between—rather no—no.

#### Identity

Identity categorization was loosely based on Heitlinger’s (2006) study with Czech and Slovak Jewish respondents. The participants were asked “How would you describe your identity?” and offered the following options: *Jewish*; *Czech*; *Jewish-Czech*; *Czech-Jewish*; *international*; *other (please specify)*. The responses could be discussed with the assessor if appropriate and then categorized in a two-stage process.

#### First stage

The researcher noted all types of identity that the respondents provided, including the options that had not been offered. The data were coded according to the originally offered categories, i.e., Jewish; Czech; Jewish-Czech; Czech-Jewish; international; other. The latter category was further specified by the respondents, for example, Jewish–Czechoslovakian, Moravian, Slovak, and others.

#### Second stage

The respondents were then divided into two groups: (A) any kind of Jewish or also Jewish identity (such as Czech-Jewish, Jewish- Czechoslovakian, etc.); and (B) without Jewish identity (e.g., Czech, European international, and others).

## Results

### Schwartz Outcome Scale-10

The sum score of SOS-10 was predicted by a separate model in each generation. To satisfy the model assumption of simple linear regression, the dependent variable was transformed using Rankit transformation (Soloman and Sawilowsky, 2009). The Group variable was coded with 1 for the focal group (survivors and their descendants) and with 2 for the comparison group. All analyses were performed using JASP v.0.14.1.0 and IBM SPSS v.23. Out of the models predicting the sum score of SOS-10, the models for the first and the second generation were significant; the first one [*F*(1, 75) = 10.18, *p* = 0.002] explained 10.8% of the outcome variance. In this model, SOS-10 was higher in the comparison group (*M* = 52.39, *SD* = 7.42) than in the focal group (*M* = 46.74, *SD* = 8.97). The model for the second generation was also significant [*F*(1, 146) = 5.49, *p* = 0.020] but explained only 3% of the outcome variance. SOS-10 was also higher in the comparison group (*M* = 50.08, *SD* = 8.41) than in the focal group (*M* = 46.71, *SD* = 9.35) (for details see Table 2).

**TABLE 2:** Prediction of SOS-10 based on the focal and comparison groups throughout generations.

Value	First generation	Second generation	Third generation
Intercept	0.29	0.37	0.32
SE	0.09	0.07	0.07
*t*	3.04	5.05	4.93
*p*	0.003	<0.001	<0.001
Group (1 = FG; 2 = CG)	0.20	0.12	0.08
SE	0.06	0.05	0.04
*t*	3.19	2.34	1.78
*p*	0.002	0.020	0.080
Observations	77	148	152
*R* ^2^	0.120	0.036	0.021
Adjusted *R*^2^	0.108	0.030	0.014
Residual std. error	0.27 (*df* = 75)	0.29 (*df* = 146)	0.26 (*df* = 150)
F statistic	10.18 (*df* = 1; 75) (*p* = 0.002)	(*df* = 1; 146) (*p* = 0.020)	3.17 (*df* = 1; 150) (*p* = 0.080)

SOS-10, Schwartz Outcome Scale; FG, Focal group; CG, Control group.

### Life satisfaction

Career satisfaction was significantly different between the focal and comparison groups only in the first (median of both groups = 5; *U* = 560.5, *p* = 0.040, r_rb_ = 0.21) and second (median of both groups = 5; *U* = 3267.5, *p* = 0.004, r_rb_ = 0.23) generations with small effect sizes. Personal life satisfaction was not different between groups in all generations (all *p* > 0.050) ($or details of proportions see Tables 3, 4).

**TABLE 3:** Sum satisfaction with career in the focal group and comparison group.

Group	% satisfied	% Rather satisfied	% Something in between	% Rather not satisfied	% Not satisfied
Focal group	72	16	5	4	3
Comparison	63	27	4	3	3

In the focal group, one percent of the data is missing.

**TABLE 4:** Sum satisfaction with personal life in the focal group and comparison.

Group	% Satisfied	% Rather satisfied	% Something in between	% Rather not satisfied	% Not satisfied
Focal group	65	17	9	4	5
Comparison	57	29	8	4	2

In the focal group, one percent of the data is missing.

### Identity

#### Focal group

Of the first generation, 74.47% of respondents (35 of 47) declared Jewish or Jewish identity of any type. More specifically, seven respondents (14.89%) identified as solely Jewish and 28 (59.57%) as Jewish or national identity of any type, i.e., 16 identified as Czech-Jewish, five as Jewish-Czech, two as Jewish-Slovak, one as Slovak-Jewish, one as Hungarian-Jewish, one as Czech-Jewish-International, one as Jewish-Czech-International, and one as Jewish-Czech-Slovak. Five of 47 respondents (10.64%) identified as solely Czech and three as other types (international, indefinite), while four were missing. This group produced 12 types of national identities altogether.

Of the second generation, 79.07% of respondents (68 of 86) declared Jewish or Jewish identity of any type. More specifically, twelve respondents (13.95%) identified as solely Jewish, and 56 (65.12%) as Jewish or national identity of any type, i.e., 28 identified as Czech-Jewish, 11 as Jewish-Czech, two as Slovak-Jewish, two as Jewish-Slovak, one as Czech-Slovak-Jewish, one as Czech and Czech-Jewish, one as Moravian-Jewish, one as Jewish-Czech-International, three as Jewish-International, one as Czech-Jewish, Jewish-Czech, International, one as Israeli, one as Israeli-Jewish, one as Jewish and Czech, one as Czech-Jewish-International, one as Jewish-Czech-International. Eleven of 86 respondents (12.79%) identified as solely Czech, one identified as solely Slovak, and one as solely Moravian, while one was missing. This group produced 19 types of national identities altogether.

Of the third generation, 66.18% of respondents (60 of 88) declared Jewish or Jewish identity of any type. More specifically, five respondents (5.68%) identified as solely Jewish, and 55 (62.5%) as Jewish or national identity of any type, i.e., 32 identified as Czech-Jewish, 12 as Jewish-Czech, one as Jewish-Slovak, one as Slovak-Jewish, one as Jewish-Czech-International, four as Jewish-International, one as Czech and Czech-Jewish, two as Czech-Jewish, and one as Slovak-Jewish-International. Sixteen respondents (18.18%) identified as solely Czech, one as Czech-International, one as Slovak, eight as International, and one as a citizen, while one was missing. This group produced 16 types of national identities altogether.

We have specifically focused on the SOS-10 results of the respondents from the focal group of the first generation who identified as solely Jewish or as Jewish and national identity of any type (i.e., Holocaust survivors with Jewish national identity). Their SOS-10 results were compared with scores of other respondents declaring other than Jewish identity (both from focal and comparison groups) from each generation.

Holocaust survivors with Jewish national identity had significantly lower average scores in SOS-10 (*M* = 46.2 ± 9.44) than other respondents with non-Jewish identity (*M* = 51.3 ± 7.83) from the first (*W* = 344, *p* = 0.014, r_rb_ = –0.360) and also from the second (with *M* = 50.2 ± 8.89, *W* = 646, *p* = 0.025, r_rb_ = –0.302), but not from the third (with *M* = 46.26 ± 8.63, *W* = 1025.50, *p* = 0.862, r_rb_ = –0.023) generations.

However, note that subsequent comparison of the SOS-10 scores of the focal group members declaring themselves as solely Jewish or mixed Jewish, with focal group respondents with non-Jewish identity in the same generation did not differ significantly in all three generations (all *p* > 0.050; see Table 5 for details).

**TABLE 5:** SOS-10 Differentiation between participants with Jewish identity and Non-Jewish identity in the focal group.

	Jewish or mixed Jewish identity				Non-Jewish identity				Mann-Whitney		
	*n*	*M*	Median	*SD*	*n*	*M*	Median	*SD*	*W*	*p*	r_rb_
First generation	25	46.20	47	9.44	15	47.53	50	9.15	160.50	0.458	–0.144
Second generation	59	45.61	47	8.92	26	48.81	52	10.05	573.50	0.065	–0.252
Third generation	57	43.21	45	9.51	31	45.03	47	9.63	766.50	0.308	–0.132

#### Comparison group

In the first generation, 15 of 31 respondents (48.39%) identified as Czechs, nine as Moravians, three as Czech-International, one as Brunensis, two as International, and one missing. This group produced five types of national identities altogether.

In the second generation, 43 of 62 respondents (69.36%) identified as Czechs, two as Moravians, two as Czech-Moravians, six as International, one as Moravian-European, one as Czech and Moravian, one as Czech-German, one as Czech-Slovak, one as European, one as Czechoslovak, one as Eastern, one as Czech-Jewish, and one as Jewish-International (the last two did not have Holocaust experiences in their family). This group produced 13 types of national identities altogether.

In the third generation, 32 of 64 respondents (50%) identified as Czechs, 10 as Moravians, eight as Internationals, three as Czech-Internationals, one as Czech-Slovak, three as Slovak, one as International-Silesian-Moravian, one as uncertain identity, one as human, one as Central European, one as Hungarian, one as Czech-European, and one as Czech-Jewish (the last one did not have any Holocaust experiences in their family). This group produced 13 types of national identities altogether.

## Discussion

In regard to psychological assessments of wellbeing and ethnic/national identity of Holocaust survivors, the territory of former Czechoslovakia represents a highly specific environment due to both its prewar history rooted in the multinational Austro-Hungarian culture and its postwar totalitarian regime enforcing, on the contrary, ethnic and social homogeneity.

### Wellbeing and satisfaction

In the first and second generations, the focal group scored lower in SOS-10 than the comparison group (*p* = 0.002 and *p* = 0.020; with small to medium effect sizes of.41 and.22, respectively), which could be interpreted as lower wellbeing of Holocaust survivors than the general population. The results reflect Barel et al.’s (2010) meta-analysis that found significantly lower wellbeing of Holocaust survivors compared to comparison groups. The wellbeing of the first generation of survivors can be dampened by unresolved emotional processes, such as mourning. According to Letzter-Pouw and Werner (2013), female survivors’ uncompleted mourning processes and their subsequent suffering of intrusive memories transmitted the emotional burden of the Holocaust to the eldest offspring and caused in them symptoms of distress. Triandis (2000) identifies variables that can lower subjective wellbeing at the cultural level, such as civil and international conflicts, political oppression, or an undemocratic government. In the Eastern/Central European countries of the postwar era, these factors, like the cold war, totalitarian regimes, political persecution including imprisonment and torture, as well as an economic decline in comparison to the West, certainly decreased the overall quality of life. The longer people had to live in the communist regime, the more negative influence they experienced on their wellbeing:

…even controlling for the fact that the formerly communist societies tend to be poorer and have larger industrial sectors than other industrial societies, the historical experience of the communist rule seems to have depressed the happiness and life satisfaction levels of the peoples who experienced it (Inglehart and Klingemann, 2000, p. 181).

Narrative responses of survivors living in Hungary, a country similar to Czechoslovakia, who were victimized during the Holocaust and subsequently experienced serial political trauma, point to a lack of social integration and ongoing threats to identity, along with fears about the rise of anti-Semitism, as factors that may adversely impact the maintenance of psychosocial wellbeing (Kahana et al., 2015). The data from the Holocaust survivors living in Hungary compared to the Holocaust survivors who immigrated to the United States or Israel after WWII show lower psychological wellbeing among survivors living in Hungary (Karady, 1993).

The model predicting the sum score of SOS-10 for the first and second generations was significant and explained 11 and 3% of the outcome variance, respectively (see Table 2). Other variables could increase the common variance. In Holocaust survivors, better health, higher instrumental coping, lower emotional coping, lesser social concern, as well as being married, having fewer life crises, communicating with co-workers, and not being resigned to fate accounted for 52% of explained variance in psychological wellbeing (Harel et al., 1988).

The absence of significant differences in the outcomes of SOS-10 of the third generation of survivors and comparisons in our study corresponds with the meta-analyses carried out by Sagi-Schwartz et al. (2003) and van IJzendoorn et al. (2003). Additionally, in Heitlinger’s (2006) study, the first generation of Czech and Slovak Holocaust survivors vacillated between being proud of their Jewishness and hiding it, while the second generation, living in the 1960s, seems to have been shedding the stigmatized identity of their parents. Berger (2010), on the other hand, maintains that the Holocaust continues to be a part of Jewish identity even in the third generation:

The third generation reveals the truth that memory and trauma, even in the face of silence, form an ineluctable part of the human experience, and that the attempt to transform the legacy of Holocaust trauma into history will, no matter the format, continue in the future (p. 158).

The focal and comparison groups did not show any significant differences in personal and career satisfaction. Similarly, Sagi-Schwartz et al. (2003) found that the Holocaust child survivors did well in their marital lives, were successful in caring for their children, and felt good about their wellbeing. A study comparing the life satisfaction of the Holocaust survivors living in Canada with native Canadians also did not reveal any significant differences between the samples, although the survivors scored lower in subjective wellbeing (Carmel et al., 2017).

### Identity

Our study results show that approximately 73.76% of the respondents in the focal group identify as fully or partly Jewish: 74.47% in the first, 79.07% in the second, and 66.18% in the third generation. The first generation of Holocaust survivors generated lesser types of national identity (12) than the second (19) and third generations (16). In the comparison group, the number of national identity types is lower: five in the first, 13 in the second, and 13 in the third generation. Eleven to 18% of the Holocaust survivors of all generations identified as exclusively Czech or Slovak (i.e., not as Jewish/also Jewish despite being of at least partly Jewish descent). The higher number of generated identity types and their combinations can also be traced in other studies. Amir and Lev-Wiesel (2001) described differences between Romanian and Polish Holocaust survivors of Jewish descent, while Romanian survivors identified mostly as Jewish and Polish survivors identified as mixed (Jewish-Polish). The authors explain the phenomenon by the different experiences of Romanian and Polish Jews during WWII, while the majority of the non-Jewish population in Romania was actively pro-Nazi, the Poles rather assumed a stance of quiet observers (which was similar in Czechoslovakia).

Of the comparison sample, 48–70% of participants identified as Czech, and the rest of the comparison group declared other or multiple identities. A similar number of the first (48%) and third (50%) generations of the comparison group opted for exclusively Czech or Slovak identity, while exclusively Czech or Slovak identification is markedly higher in the second generation of non-survivors (70%), most probably because the second generation had lived in the totalitarian regime intolerant to minorities and any kind of otherness. It could be speculated that the Jewish subpopulation, despite the state anti-Semitism and overall tendency to achieve ethnic homogeneity, was able to resist the totalitarian oppression in this sense more than the majority, thus generating more types of ethnic identities even today.

In this study, we explored the categories and content of ethnic/national identity and not its intensity. There are, however, studies assessing the intensity of identity in another major local ethnic minority, the Roma. The association between Roma identity and wellbeing was positive in the Czech Republic and Romania, but negative in Kosovo and Bulgaria. In the Czech Republic, the Roma showed a lower endorsement of national identity but stronger religious identity than their majority counterparts (Dimitrova et al., 2017). According to Stejskalová (2012), the Roma ethnic group experience higher levels of non-inclusivity in the Czech society than the Jewish population.

Researchers found a positive correlation between ethnic identity and psychological wellbeing and psychosocial adjustment in different samples (Smith and Silva, 2011; Rivas-Drake et al., 2014). In eight studies of wellbeing in youth, wellbeing positively influenced the adjustment of an ethnic subgroup (*r* = 0.20, *p* < 0.050; Rivas-Drake et al., 2014). A meta-analysis of 184 studies found that ethnic identity was modestly related to positive wellbeing (Smith and Silva, 2011). Different aspects of wellbeing, i.e., measures of self-esteem, self-mastery, and general wellbeing yielded the largest average effect sizes, and measures of mental health symptoms yielded the smallest average effect sizes (Smith and Silva, 2011).

In our focal sample, participants of all three generations with Jewish or Jewish identity of any type scored lower in SOS-10 (significantly only in G1), that is, their wellbeing was lower than in the survivors with non-Jewish identity. Jewish self-identification, even if, as in our study, the identity is declared in a safe xenophile environment, could lower wellbeing probably because of the experience of otherness, feelings of alienation from the majority population, especially in the first and second generations of survivors, and fear of negative consequences for the individual and their family should their identity be publicized. War experiences could strongly modify identity formation to “…the extent to which acquired survival strategies consisting of pretending to be someone else affected these people’s post-war identities” (Prot, 2008, p. 243).

### Strengths and limitations of the study

The presented study is one of a few studies exploring subjective wellbeing in relation to the national identity of Holocaust survivors that is conducted in post-communist Eastern/Central European countries. SWB assessment is based on just one self-report measure (SOS-10) and two brief questions regarding life satisfaction, without seizing more detailed methods of assessment regarding the quality of life, such as the Satisfaction with Life Scale (Diener et al., 1985). Identity was assessed only categorically, revealing a wide variety of identity types in Czech and Slovak Holocaust survivors. Instruments measuring the intensity of identity, such as the Anchor Periods Questionnaire (Shmotkin et al., 2006) or the centrality of an event to individual identity (e.g., Berntsen and Rubin, 2006), however, were not employed in the study even if event centrality is a distinctive mechanism in intergenerational transmission in survivor families (Greenblatt-Kimron et al., 2021). The number of respondents in the subgroups would not enable a representative interpretation of the results. Regarding the control group G1, it is uncertain whether or not members of this group were exposed to any hardships during the German occupation, such as fear or hunger. Also, the three generations were not biologically related.

## Conclusion

The study compared the wellbeing and national identity of three generations of Czechoslovak Holocaust survivors with aged-matched controls. The first and second generations of survivors showed lower wellbeing than the general population. The wellbeing of the third generation of survivors, as well as the personal and career satisfaction of all three generations of survivors, do not differ significantly from comparisons. Holocaust survivors of all three generations identify as solely Jewish or as Jewish identity of any type in 73–79% of cases; their Jewish identity is, however, mostly mixed with other national identities present in the post-Czechoslovakian territory. Of the Holocaust survivors in all three generations, 11–18% identify as exclusively Czech or Slovak, completely denying their Jewish descent. Holocaust survivors identifying as Jewish or also Jewish show lower wellbeing than survivors denying their Jewishness.

All cohorts, focal as well as comparisons, generated more types of ethnic/national identity than would be expected in a comparatively small sample from an allegedly ethnically homogenous society. The complex questions of ethnic/national identity and its psychological impact on both Jewish and non-Jewish populations in the Central European space with its Austro-Hungarian historical background and post-communist legacy deserve a profound exploration that is beyond the scope of this study.

## Data availability statement

The raw data supporting the conclusions of this article will be made available by the authors, without undue reservation.

## Ethics statement

The study was conducted in accordance with the Declaration of Helsinki, and the protocol was approved by the Ethical Committee of the National Institute of Mental Health (number 48/16). The patients/participants provided their written informed consent to participate in this study.

## Author contributions

MP: data collection, statistical analysis, repeated reading of the manuscript, and management of the author’s team. MF and MN: data collection, organization of data collection, and statistical analysis. RH: statistical analysis, methdological contributions, and repeated reading of the manuscript. PB and AP: data collection and discussion of the manuscript. DŠ: repeated reading of the manuscript. ES: proof-reading and repeated reading of the manuscript. All authors contributed to the article and approved the submitted version.
